# Parental microbiome programming of early-life neurodevelopment: multi-niche contributions through the microbiome–gut–brain axis

**DOI:** 10.1080/19490976.2026.2673888

**Published:** 2026-05-14

**Authors:** Jurga Skrabulyte-Barbulescu, Lidya K. Yassin, Saif Almazrouei, Shamma H. Alkuwaiti, Sultan Almarzooqi, Fatima Alnuaimi, Salma Alketbi, Mohammed M. Nakhal, Paulina Rutkowska-Gauvry, Suhail A. Matar, Mohammad I.K. Hamad

**Affiliations:** a The Institute of Psychiatry, Psychology and Neuroscience (IoPPN), King´s College London, London, United Kingdom; b Department of Anatomy, College of Medicine and Health Sciences, United Arab Emirates University, Al Ain, United Arab Emirates; c Department of Surgery, College of Medicine and Health Sciences, UAE University, Al Ain, United Arab Emirates

**Keywords:** Microbiota–gut–brain axis, early-life neurodevelopment, maternal microbiome, paternal microbiome, vertical microbial transmission, short-chain fatty acids, microbial metabolites, perinatal programming

## Abstract

The microbiota–gut–brain axis (MGBA) is a central pathway through which gut microbial communities influence neurodevelopment via immune, metabolic, and neural signalling. Early life, spanning preconception through infancy, represents a particularly sensitive window during which parental microbiomes exert disproportionate influence on offspring gut colonization, immune education, and neurodevelopmental programming. This review synthesizes current evidence on how maternal and paternal microbiomes shape pediatric neurodevelopment through coordinated microbial, metabolic, immune, and epigenetic pathways. We examine pregnancy-associated remodeling of maternal microbiomes across gut, vaginal, oral, skin, and milk niches, highlighting how hormonal, metabolic, and immune adaptations drive site-specific microbial shifts with downstream consequences for fetal and infant brain development. Core microbial mechanisms are discussed, including short-chain fatty acids (SCFAs), tryptophan-derived metabolites, bile-acid signaling, and immune mediators that link microbial metabolism with immune and neurodevelopmental processes. These mechanisms are integrated with key transmission routes, including placental metabolite transfer, mode-of-delivery–dependent microbial seeding, breast milk–mediated signaling, and early environmental exposures that further shape the developing MGBA. We also incorporate emerging evidence on paternal microbiome contributions via preconception programming, sperm epigenetic remodeling, and germline–microbiome interactions, expanding the traditional maternal-centric view of intergenerational microbial inheritance. Finally, we evaluate modifiable factors, including diet, metabolic status, stress, antibiotic exposure, and microbiome-targeted interventions, and discuss their translational relevance. While associations between the microbiome and neurodevelopment are increasingly supported by human studies, many mechanistic insights remain derived from animal models, and causal relationships are not yet fully established. By integrating mechanistic, clinical, and systems-level perspectives, this review positions the MGBA as a promising but still evolving framework for understanding and potentially modulating early-life brain development.

## 1. Introduction

**Figure 1.**
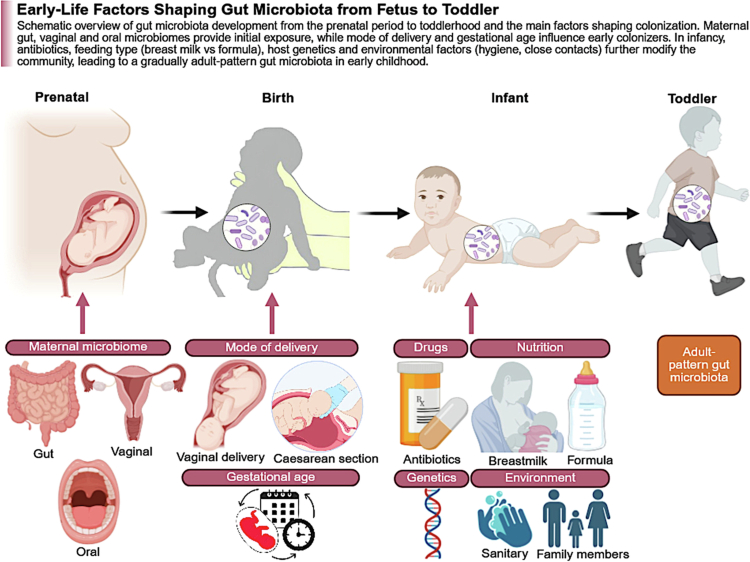
Early-life factors shaping gut microbiota from fetus to toddler. Schematic overview of gut microbiota development from the prenatal period to early childhood, highlighting key factors that influence microbial colonization across developmental stages. Maternal microbiomes (gut, vaginal, and oral) contribute to initial microbial exposure, while mode of delivery and gestational age shape early colonization at birth. During infancy, antibiotic exposure, feeding type (breast milk versus formula), host genetics, and environmental factors (e.g., hygiene and close contacts) further modulate microbiota composition, leading to gradual maturation toward an adult-like gut microbiome. (Created with BioRender).

The MGBA is a bidirectional communication network through which intestinal microbes and their metabolites influence neural, immune, endocrine, and metabolic signaling.^1-5^ Early life, spanning preconception, pregnancy, birth, and infancy, represents a particularly sensitive developmental window during which microbial signals shape immune maturation, neurogenesis, synaptogenesis, and later behavioral trajectories.^6-10^ Microbial colonization during this period is not a single event but a dynamic, staged process influenced by parental microbiomes, delivery mode, early-life exposures, and environmental factors, all of which converge to shape the developing MGBA. This staged progression of microbial colonization across prenatal life, birth, infancy, and early childhood is summarized in Figure 1. During this window, maternal microbiomes act as primary drivers of offspring microbial assembly through metabolite transfer during pregnancy and direct microbial transmission at and after birth, while emerging evidence suggests that paternal microbiome-related effects may also contribute through preconceptional influences on sperm epigenetic programming and reproductive signaling.^11-13^ However, several key controversies remain unresolved, including whether maternal effects are mediated predominantly by live microbial transfer or by metabolites and immune signals, the strength of causal evidence linking microbiomes to neurodevelopment, and the extent to which findings from animal models translate to humans.^6^^,^^14^^,^^15^

This review examines how parental microbiomes influence early-life neurodevelopment across the MGBA, with emphasis on maternal gut, vaginal, milk, oral, and skin niches and a focused discussion of paternal gut and seminal microbiome-related contributions. Rather than considering these niches independently, we frame them as temporally coordinated sources of microbial, metabolic, and immune signals that act across gestation, delivery, lactation, and early postnatal life.^16^^,^^17^ We first define core microbial mechanisms relevant to neurodevelopment, then examine the major transmission pathways linking parental microbiomes to the offspring gut and brain, followed by modifiable factors that disrupt or support these processes. Finally, we address paternal contributions and highlight translational opportunities and key evidence gaps. By integrating maternal multi-niche biology with emerging paternal perspectives, this review proposes a unified framework in which parental microbiomes collectively shape early neurodevelopment through interconnected microbiome–brain axes.^12^^,^^13^^,^^18^^,^^19^

## 2. Maternal microbiome: composition and physiological context

### 2.1. Introduction

Pregnancy and lactation are accompanied by coordinated endocrine, immune, and metabolic adaptations that reshape the maternal microbiome across multiple body sites, including the gut, vagina, oral cavity, skin, and milk.^20-22^ These microbial ecosystems do not change uniformly. Rather, each niche responds differently to host physiology, environmental exposures, and clinical factors, with implications for maternal health, birth outcomes, and early microbial transfer to the infant.^23^^,^^24^ Among these niches, the maternal gut microbiota shows the most extensively characterized remodeling during pregnancy, particularly in late gestation, when metabolic and inflammatory adaptations intensify.^25^^,^^26^ However, the vaginal, oral, skin, and milk microbiomes are also relevant because they represent potential routes through which maternal physiology and perinatal exposures may influence early microbial inheritance.^27-29^ Importantly, this section focuses on how these maternal niches change across pregnancy and lactation and on the factors that modulate them. The neurodevelopmental relevance of specific microbial taxa and transmission pathways is examined in Section 3.

### 2.2. Physiological adaptations during pregnancy and lactation

Pregnancy is characterized by tightly regulated hormonal, metabolic, and immune adaptations that support implantation, placentation, fetal growth, and preparation for parturition and lactation, while also shaping microbial ecology at maternal mucosal surfaces.^22^^,^^30^ Human chorionic gonadotropin becomes detectable within days of fertilization and supports progesterone and estrogen production, both of which rise substantially across gestation and influence epithelial integrity, immune tone, and microbial composition.^30^^,^^31^ These hormones also affect the gut–brain axis indirectly by modulating intestinal barrier function, mucosal immunity, and bacterial growth conditions.^32^ Maternal metabolism likewise shifts across gestation. Early pregnancy is relatively anabolic, favoring energy storage, whereas mid-to-late pregnancy becomes increasingly catabolic, with reduced insulin sensitivity and greater mobilization of glucose and lipids to support fetal growth.^33^^,^^34^ Although these changes are physiological, they resemble some features of metabolic syndrome and provide an important context for understanding pregnancy-associated microbiome remodeling.^33^^,^^35^

Immune regulation also changes over time. Early gestation is relatively pro-inflammatory to support implantation; mid-gestation shifts toward immune tolerance; and late gestation and parturition are again associated with inflammatory activation.^36^^,^^37^ These stage-dependent immune shifts likely contribute to the restructuring of maternal microbial communities across body sites.^38^ After delivery, the sharp decline in progesterone and estrogen permits prolactin-driven milk synthesis, while oxytocin supports milk ejection in response to suckling.^39^^,^^40^ Lactation also imposes major energetic demands and introduces a distinct mammary immune environment rich in secretory IgA and other host factors relevant to neonatal microbial colonization.^41^ Together, these physiological transitions provide the host context in which maternal microbiomes are remodeled during pregnancy and postpartum.

### 2.3. Maternal gut microbiota during pregnancy

The maternal gut microbiota undergoes progressive, trimester-dependent remodeling during pregnancy, although the magnitude and direction of change vary across cohorts and study designs.^25^^,^^26^ In healthy pregnancy, commonly reported taxa include Bifidobacterium, Blautia, Bacteroides, Akkermansia, Faecalibacterium, Ruminococcus, and members of the Lachnospiraceae, Ruminococcaceae, and Clostridiales.^22^ During the first trimester, gut microbial composition often resembles that of non-pregnant women, with relatively greater abundance of butyrate-producing taxa such as Faecalibacterium and Eubacterium and higher alpha diversity in some cohorts.^25^^,^^42^ Between the first and second trimesters, compositional changes become more apparent, with alterations in beta diversity indicating that community structure is beginning to shift even when broad phylum-level patterns remain stable.^43^^,^^44^ In a longitudinal U.S. cohort, gut microbial beta diversity differed between the first and second trimesters but not between the second and third, suggesting that part of this remodeling may stabilize by mid-pregnancy.^43^ Data from Chinese cohorts also indicate second-trimester enrichment of *Firmicutes*, *Bacteroidetes*, *Actinobacteria*, *Tenericutes*, and *Proteobacteria*, with increased representation of *Bifidobacteriaceae* and *Enterobacteriaceae.*^45^ The third trimester is the most frequently studied stage and is often characterized by reduced alpha diversity, increased inter-individual variation in community composition, and enrichment of taxa associated with inflammation or altered metabolic status, including Proteobacteria, Actinobacteria, Enterobacteriaceae, Streptococcus, and Collinsella.^25^^,^^46^^,^^47^ At the same time, some studies report increased abundance of *Bifidobacterium* and *Blautia* in late pregnancy, suggesting that late-gestation remodeling does not simply reflect loss of beneficial taxa but rather a complex ecological reorganization shaped by host physiology.^48^ Progesterone may contribute directly to this process, as experimental supplementation in non-pregnant mice increased Bifidobacterium abundance in vivo and in vitro.^48^ Hormonal effects may also act indirectly through immune and metabolic changes that alter the intestinal environment.^26^^,^^49^ Overall, the maternal gut microbiota appears to shift across pregnancy toward a less diverse and more individualized late-gestation configuration, often with greater representation of lactic-acid-producing and inflammation-associated taxa.^25^^,^^47^ However, interpretation should remain cautious. Findings vary across studies because of differences in sampling time, cohort characteristics, antibiotic exposure, dietary patterns, body composition, and sequencing approaches, and many studies remain underpowered or focused primarily on late pregnancy.^23^ This variability is important and should be acknowledged rather than treated as inconsistency to be ignored.

### 2.4. Vaginal microbiome

The vaginal microbiome plays a central role in reproductive health and is one of the best-defined maternal microbial niches relevant to pregnancy. In healthy reproductive-age women, vaginal communities are often dominated by Lactobacillus species, which promote a low pH through lactic acid production and thereby restrict pathogen growth.^50^^,^^51^ During pregnancy, the vaginal microbiome generally becomes more stable and more likely to remain *Lactobacillus*-dominant than in the non-pregnant state.^27^^,^^51^ Community state types are commonly classified according to dominant taxa, with four *Lactobacillus*-dominated states and a fifth enriched in anaerobes with lower lactic acid production.^52^ Pregnancy favors the former pattern. Longitudinal work has shown that pregnant women who deliver at term more often exhibit stable communities enriched in *Lactobacillus crispatus*, *L. gasseri*, *L. jensenii*, and related taxa, while bacterial-vaginosis-associated genera such as *Prevotella*, *Sneathia*, *Gardnerella*, *Mobiluncus*, and *Parvimonas* are less prevalent.^27^^,^^53^ These shifts are thought to be influenced in part by rising estrogen levels, which promote glycogen availability and thereby support Lactobacillus metabolism and lactate production.^53^

Evidence also suggests that failure to maintain early-pregnancy vaginal stability is associated with adverse outcomes, particularly preterm birth. In one study, women who later delivered preterm showed significant reductions in richness, diversity, and evenness between the first and second trimesters, whereas community structure remained relatively stable in term pregnancies.^54^ Other cohorts similarly report that early gestation may be a critical window during which vaginal microbiome organization is linked to pregnancy outcome.^55^ At the same time, the vaginal microbiome is shaped by a range of biological and social factors, including age, reproductive history, sexual behavior, hygiene practices, and ancestry, which may contribute to between-cohort heterogeneity and should not be oversimplified as purely biological variation.^56-58^ Taken together, current evidence supports the view that pregnancy is generally associated with a stable, low-diversity, Lactobacillus-enriched vaginal microbiome, and that loss of this stability may mark vulnerability to adverse obstetric outcomes.^27^^,^^54^ The neurodevelopmental implications of vaginally transferred taxa are considered later in the manuscript.

### 2.5. Oral microbiome

The oral cavity hosts one of the largest and most diverse microbial communities in the body, comprising more than 700 species across multiple oral habitats.^59^^,^^60^ Pregnancy is associated with measurable changes in oral microbial load and community composition, likely driven by hormonal fluctuation, altered immune tone, and increased susceptibility to gingival inflammation.^60^ Clinical studies suggest that pregnancy, particularly the first and second trimesters, is associated with enrichment of periodontal-associated and opportunistic taxa, including *Porphyromonas gingivalis*, *Aggregatibacter actinomycetemcomitans*, *Streptococcus*, *Staphylococcus*, and *Candida* species.^61^ Elevated progesterone appears to increase susceptibility to plaque-associated gingival inflammation, and the abundance of some oral pathobionts has been positively correlated with circulating hormone levels during gestation.^62^ Additional taxa linked to periodontal dysbiosis, such as Prevotella intermedia, Campylobacter rectus, and Prevotella nigrescens, have also been associated with pregnancy-related hormonal changes.^60^ These oral shifts are clinically relevant because periodontal disease and oral dysbiosis during pregnancy have been associated with preterm birth, preeclampsia, and low birth weight, although causality remains difficult to establish and shared inflammatory pathways may contribute to these associations.^63^^,^^64^ Importantly, oral microbial alterations appear at least partly reversible: postpartum, total bacterial load and some pathogenic taxa decline toward a non-pregnant baseline.^65^ Thus, current evidence supports the conclusion that the oral microbiome is a pregnancy-responsive niche whose dysregulation may reflect broader host inflammatory and hormonal changes.

### 2.6. Skin microbiome

Compared with the gut, vagina, and oral cavity, the maternal skin microbiome remains less well characterized during pregnancy. Available evidence nevertheless suggests that pregnancy is associated with structured reorganization of skin microbial communities, particularly in late gestation.^29^ In the first study to profile skin microbiota across trimesters, microbial composition differed across all pregnancy stages compared with non-pregnant controls, with the greatest divergence observed in the third trimester.^29^ Late pregnancy was associated with increased abundance of *Pseudomonas* and *Janthinobacterium* and reduced abundance of taxa such as *Sphingomonas* and *Oscillospira*, alongside evidence of altered network centrality for *Corynebacterium simulans*, a species associated with healthy skin microbial communities.^29^ These findings suggest that pregnancy may affect not only relative taxonomic abundance but also the ecological organization of the skin microbiome. However, this remains a relatively underdeveloped area. The current evidence base is small, often cross-sectional, and insufficient to determine whether observed changes are generalizable across populations or body sites. For that reason, the skin microbiome should be included in the review because of the manuscript’s multi-niche scope, but discussed proportionately and cautiously.

### 2.7. Milk microbiome

**Figure 2.**
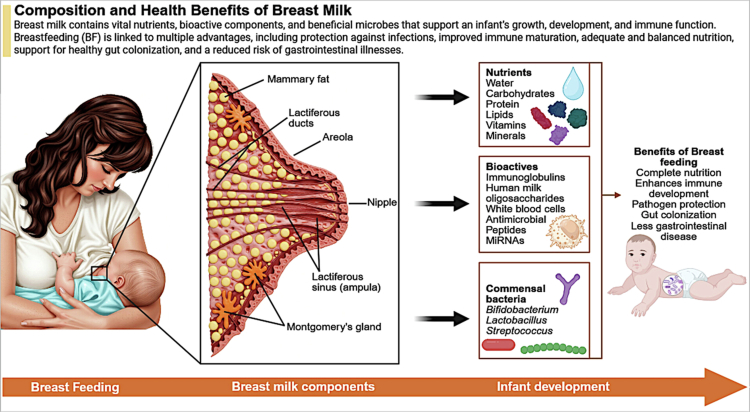
Composition and functional role of breast milk in infant development. Schematic representation of the major components of human breast milk and their contributions to infant growth, immune maturation, and gut colonization. Breast milk contains macronutrients (carbohydrates, lipids, proteins), micronutrients (vitamins and minerals), bioactive compounds (immunoglobulins, human milk oligosaccharides, antimicrobial peptides, and microRNAs), and commensal bacteria, including *Bifidobacterium*, *Lactobacillus*, and *Streptococcus*. These components collectively support intestinal barrier function, immune development, pathogen protection, and early microbiota establishment, with downstream implications for neurodevelopment. Abbreviations: HMO, human milk oligosaccharides; miRNA, microRNA. (Created with BioRender).

Human milk is a complex biological system that contains nutrients, immune mediators, bioactive molecules, and a dynamic microbial community that changes across lactation.^66^^,^^67^ The major nutritional, bioactive, and microbial components of breast milk and their relevance to infant development are summarized in Figure 2. The origin of milk-associated microbes remains debated, with likely contributions from maternal skin, the infant oral cavity, environmental exposure, and possibly the maternal gut via an entero-mammary route.^67^^,^^68^ Regardless of origin, milk represents an important postnatal microbial and biochemical interface between mother and infant. Milk microbial composition varies by lactation stage. Colostrum generally has the highest microbial diversity and may provide a broad early inoculum, with reported enrichment of Firmicutes and genera such as *Staphylococcus*, *Lactobacillus*, *Acinetobacter*, and *Pseudomonas.*^69^^,^^70^ Strain-level work also suggests that early milk contains diverse Bifidobacterium populations relevant to infant gut colonization.^68^ As lactation progresses, community structure shifts. Transitional milk is associated with rapid remodeling, while mature milk shows changing relative abundance of *Bifidobacterium*, *Lactobacillus*, *Pseudomonas*, and *Streptococcus*, with some core genera remaining detectable throughout lactation.^28^^,^^69^ Later lactation may increasingly reflect bidirectional mother–infant exchange, as oral-associated taxa such as *Actinomyces*, *Veillonella*, *Rothia*, and *Prevotella* become more abundant, consistent with retrograde transfer from the infant oral cavity during breastfeeding.^28^ At the same time, milk microbiome trajectories are not identical across populations. Studies from Irish, Chinese, and Thai-Myanmar cohorts suggest that geography, host characteristics, and environment influence lactational microbial succession.^28^^,^^69^^,^^71^ Preterm birth may also alter milk microbial organization, with enrichment of taxa such as *Staphylococcus haemolyticus*, *Propionibacterium acnes*, and gut-associated bacteria compared with term milk.^71^ Overall, human milk should be viewed not as a static fluid but as a dynamic microbial niche whose composition changes across lactation and may contribute to early-life microbial programming.

### 2.8. Key modulators of the maternal microbiome

Multiple maternal exposures can remodel microbial communities during pregnancy and lactation. To reduce overlap with later mechanistic and intervention-focused sections, the emphasis here is on how these factors alter maternal microbiomes rather than on repeating downstream offspring outcomes in detail.

#### 2.8.1. Maternal obesity

Maternal overweight and obesity are consistently associated with altered microbial composition across multiple maternal niches, particularly the gut, vagina, and milk.^43^^,^^72^^,^^73^ In the gut, pre-pregnancy overweight and obesity have been linked to enrichment of pro-inflammatory or bile-tolerant taxa such as *Bilophila* and depletion of taxa associated with metabolically favorable functions, including *Phascolarctobacterium*.^43^ These associations often correlate with maternal body mass index (BMI), fat mass, insulin, and triglyceride levels, suggesting that microbial shifts occur within a broader metabolic context rather than in isolation. Obesity is also associated with altered vaginal microbial profiles. Women with higher BMI are more likely to show reduced Lactobacillus dominance, greater community diversity, and enrichment of taxa associated with bacterial vaginosis, including *Prevotella.*^72^^,^^74^^,^^75^ The mechanisms linking obesity to vaginal dysbiosis remain uncertain but may involve hormonal, immune, metabolic, and gut–vaginal ecological interactions.^76^^,^^77^ In addition, obesity has been associated with reduced abundance of beneficial taxa such as *Bifidobacterium* in mature milk, suggesting that maternal metabolic status may shape postnatal microbial exposure through the milk niche as well.^73^ Overall, obesity should be considered a major maternal microbiome modulator, but current evidence remains stronger for association than mechanism. Many studies are cross-sectional or limited by single sampling points, making it difficult to distinguish stable obesity-related signatures from pregnancy-stage effects or dietary confounding.

#### 2.8.2. Prenatal stress

Prenatal stress is associated with measurable changes in maternal gut, vaginal, and milk microbiomes, although findings are heterogeneous and likely depend on the timing and type of stress exposure assessed.^47^^,^^78-80^ Stress activates the hypothalamic–pituitary–adrenal (HPA) axis and elevates circulating glucocorticoids and catecholamines, which may influence microbial ecology indirectly through host physiology and, under some conditions, directly through bacterial growth responses.^81^ In human pregnancy cohorts, perceived stress has been associated with reduced gut alpha diversity and with trimester-specific shifts in community structure, including enrichment of taxa such as *Faecalitalea*, *Catenibacterium*, *Prevotella*, and *Streptococcus pasteurianus* in different gestational windows.^47^^,^^78^ Stress and depressive symptoms have also been linked to altered vaginal diversity trajectories, although large compositional disruptions are not consistently observed, which is in keeping with the relative stability of the vaginal niche during healthy gestation.^27^^,^^78^ Emerging data further suggest that stress may influence the milk microbiome postpartum. Mothers exposed to higher early postpartum stress showed reduced relative abundance of *Streptococcus*, *Gemella*, and *Veillonella* and enrichment of *Staphylococcus*, *Corynebacterium*, and *Acinetobacter* in milk samples collected during the first month postpartum.^79^ A randomized trial also found that stress-reduction interventions in lactating mothers were associated with increased Bifidobacterium abundance in breast milk.^82^ Taken together, these findings support the view that psychological stress is a plausible cross-niche modulator of the maternal microbiome, although directionality and persistence remain incompletely resolved.

#### 2.8.3. Maternal diet

Maternal diet is one of the most modifiable influences on the microbiome during pregnancy and lactation and affects multiple maternal niches, especially the gut and milk microbiomes.^83^^,^^84^ In the gut, plant-rich or vegetarian dietary patterns have been associated with greater abundance of taxa linked to SCFA production, including *Roseburia* and *Lachnospiraceae*, and lower abundance of *Collinsella*, which has been associated with adverse metabolic profiles.^83^ In contrast, obesogenic diets in animal models have been linked to reductions in *Lactobacillus* and *Romboutsia* and to altered micronutrient status, suggesting that diet can affect both microbial ecology and metabolic output.^85^ Dietary effects also extend beyond the gut. In a large U.S. pregnancy cohort, higher intake of low-fat dairy, fruit, and fiber was associated with increased likelihood of a *Lactobacillus crispatus*-dominant vaginal microbiome, whereas other dietary patterns were linked to less favorable community states.^86^ Similarly, pre-pregnancy carbohydrate intake has been associated with higher vaginal Lactobacillus abundance, whereas higher animal protein intake has been linked to vaginal dysbiosis in smaller cohorts.^72^ In milk, maternal intake of carbohydrates, lipids, polyphenols, plant proteins, and fiber has been associated with variation in the abundance of *Bifidobacterium*, *Staphylococcus*, and *Veillonella*, among other taxa, although most available studies are observational and cross-sectional.^67^ Thus, current evidence supports maternal diet as a multi-niche microbial modulator, but stronger longitudinal and mechanistic studies are needed before dietary signatures can be interpreted as causal drivers of maternal microbial programming.

#### 2.8.4. Antibiotic exposure

Antibiotic exposure during pregnancy is common and can disrupt maternal microbial communities across gut, vaginal, and milk niches.^87-90^ In the vagina, intrapartum penicillin administration for Group B *Streptococcus* prophylaxis has been associated with marked reductions in *Lactobacillus* dominance, indicating a substantial perturbation of the protective vaginal community.^87^ In the gut, experimental vancomycin exposure in pregnant mice reduced maternal alpha diversity, depleted short-chain-fatty-acid-producing taxa, and enriched genera associated with inflammation and impaired barrier function.^89^ Milk microbiota may also be affected. In one Finnish cohort, Bifidobacterium was detected in breast milk at one month postpartum only among mothers who had not received intrapartum antibiotics, suggesting that antibiotic exposure may influence the transfer or persistence of beneficial milk-associated taxa.^88^ However, studies in this area remain limited, and some report increased diversity rather than simple depletion, underscoring the need for more nuanced interpretation.^88^ Overall, antibiotic exposure should be treated as a major perturbing factor rather than a uniform cause of “bad” microbiota. The magnitude, duration, and clinical consequences of microbiome disruption likely depend on the timing, spectrum, and indication for antibiotic use, as well as on host and environmental context.

## 3. Core microbial mechanisms in neurodevelopment

Early-life neurodevelopment is influenced not by the maternal microbiome in general, but by a more limited set of vertically transmitted or maternally shaped microbial groups and their metabolites. To orient the reader before discussing transmission routes, this section summarizes the principal microbial mechanisms most relevant to offspring neurodevelopment across the MGBA. The central concept is that maternal microbial communities contribute to neurodevelopmental programming through three broad and interacting functional categories: beneficial commensals, which support barrier maturation, immune calibration, and neuroactive metabolite production; pathobionts, which under dysbiotic conditions amplify inflammatory and permeability-related signaling; and context-dependent taxa, whose effects vary by strain, ecological background, and host metabolic state.^18^^,^^91^ Mechanistically, the microbial signals most consistently implicated in neurodevelopment include SCFAs, tryptophan-derived metabolites, bile-acid derivatives, vitamins involved in one-carbon metabolism, and immune-modulating microbial products that influence placental signaling, microglial maturation, BBB integrity, synaptogenesis, and postnatal gut colonization.^15^^,^^92-95^ In addition to these processes, extracellular signaling pathways that regulate dendritic architecture and neuronal network maturation are critical components of early brain development.^96-98^Importantly, the strength of evidence differs across pathways. Associations between maternal and infant microbial profiles are increasingly supported by human strain-tracking studies, whereas many mechanistic links to offspring brain development remain strongest in animal or translational models rather than in direct human causal studies.^6^^,^^17^^,^^99^^,^^100^

### 3.1. Beneficial commensals and neurodevelopment-supportive signaling

Among the taxa most consistently linked to healthy early-life programming are *Bifidobacterium*, *Bacteroides*, *Lactobacillus*, and several SCFA-producing anaerobes, including *Faecalibacterium*, *Roseburia*, *Subdoligranulum*, *Anaerobutyricum hallii*, and related Lachnospiraceae and Ruminococcaceae members.^91^^,^^101^ These taxa are relevant because they occupy complementary ecological roles rather than acting in isolation. *Bifidobacterium* is a key early-life commensal because it is strongly enriched in breastfed infants, can be vertically transmitted from mother to infant, and is closely linked to human milk oligosaccharide utilization, acetate and lactate production, mucosal barrier support, and immune maturation.^102^ Experimental work further suggests that bifidobacteria and their metabolites can shape synapse formation, microglial function, and neurotrophin-related signaling, although these mechanistic data derive mainly from preclinical models.^103^^,^^104^ Human observational studies are directionally consistent, linking higher early-life *Bifidobacterium* abundance with more favorable inflammatory and developmental profiles, but they remain largely associative.^105^

*Bacteroides* should also be established early as a core neurodevelopmentally relevant genus because it is central to fecal vertical transmission and infant gut ecological succession. Vaginally delivered infants are more likely to acquire maternal *Bacteroides* strains, whereas cesarean delivery is associated with reduced early *Bacteroides* abundance and delayed microbiome maturation.^16^^,^^106^^,^^107^ Functionally, *Bacteroides* contributes to complex glycan degradation, cross-feeding networks, immune education, and SCFA-related metabolism, thereby helping establish an intestinal environment that supports barrier integrity and downstream gut–brain signaling.^11^^,^^108^ Although direct human proof that maternally transmitted *Bacteroides* improves neurodevelopment is still limited, its repeated association with healthier colonization trajectories makes it a foundational taxon in the developmental narrative.

*Lactobacillus* is particularly important in relation to the vaginal microbiome, delivery, and breast milk. During healthy pregnancy, *Lactobacillus*-dominated vaginal communities are associated with stability and protection from dysbiosis, and vaginal delivery provides an important route through which lactobacilli contribute to initial neonatal seeding.^27^^,^^55^ *Lactobacillus* also appears in milk and infant gut ecosystems and has recognized immunomodulatory and anti-inflammatory functions, including lactate production, pathogen exclusion, and support of epithelial homeostasis.^68^^,^^109^ Some preclinical data further suggest that specific *Lactobacillus* species may influence offspring social behavior, vagal signaling, and stress-related pathways, but these findings remain strain-specific and should not be generalized to the entire genus.^110^^,^^111^

Beyond these early colonizers, several strict anaerobic SCFA producers are likely to support neurodevelopment indirectly by generating butyrate, propionate, and acetate. Taxa such as *Faecalibacterium prausnitzii*, *Roseburia hominis*, *Subdoligranulum*, and *Anaerobutyricum hallii* contribute to anti-inflammatory signaling, epithelial barrier maintenance, and metabolic cross-feeding networks that sustain a mature and resilient gut ecosystem.^112-114^ Butyrate is especially relevant because it has been linked to histone deacetylase inhibition, neurotrophin regulation, microglial maturation, and synaptic plasticity in experimental systems.^92^^,^^115^ However, in human pregnancy and infancy, the neurodevelopmental significance of these taxa is still inferred primarily from metabolite biology, ecological associations, and animal studies rather than from direct interventional evidence. Taken together, the most plausible model is that beneficial commensals support offspring neurodevelopment not because any single genus is uniquely protective, but because vertically transmitted maternal taxa help establish a metabolically cooperative early-life ecosystem enriched for SCFA production, glycan utilization, immune tolerance, and barrier stabilization. These processes are likely more biologically robust than taxon-by-taxon claims.

### 3.2. Pathobionts and neurodevelopment-disruptive signaling

In contrast, maternal dysbiosis may increase offspring neurodevelopmental vulnerability by enriching taxa that promote inflammation, epithelial disruption, and aberrant immune signaling. The term pathobiont is used here to describe organisms that are not universally harmful but may become detrimental when ecological balance is disturbed or when host inflammatory conditions favor pathogenic behavior.^116^ Among the taxa most often implicated are *Bilophila wadsworthia*, *Desulfovibrio*, opportunistic *Escherichia coli* pathotypes, and other inflammation-associated Proteobacteria or sulfide-producing organisms.^117-119^ Their relevance lies less in simple presence than in the types of signals they amplify, including lipopolysaccharide, hydrogen sulfide, oxidative stress, impaired tight-junction integrity, and pro-inflammatory cytokine cascades. These mechanisms have been linked experimentally to increased gut permeability, altered BBB function, microglial activation, and impaired synaptic development.^108^^,^^120^^,^^121^ For example, expansion of *Bilophila* has been associated with inflammatory states and maternal obesity-related dysbiosis, while *Desulfovibrio* has been linked to sulfur metabolism, barrier dysfunction, and inflammatory neuroimmune signatures in disease-oriented studies.^43^^,^^117^^,^^118^ Similarly, vertically transmitted or perinatally acquired *E. coli* pathotypes may contribute to neonatal inflammatory burden through lipopolysaccharide and other virulence-associated products, particularly in vulnerable infants.^122-124^ However, evidence linking these taxa specifically to later neurodevelopment remains much stronger in mechanistic and animal studies than in human longitudinal cohorts. A more accurate interpretation is therefore that pathobiont-enriched maternal dysbiosis may create a pro-inflammatory developmental context that plausibly influences fetal and infant neurodevelopment, rather than that specific organisms directly cause neurodevelopmental disorders.

### 3.3. Context-dependent taxa and strain-level effects

A third group includes taxa whose developmental significance is context dependent, meaning that their effects vary according to strain, maternal metabolic state, surrounding microbial ecology, and timing of exposure. This distinction is important because genus-level classification alone often obscures functional diversity. *Akkermansia muciniphila* illustrates this concept. In some contexts, it is associated with improved barrier function, metabolic health, and anti-inflammatory signaling, whereas in others excessive mucin degradation or strain-level variation may contribute to barrier fragility or inflammatory responses.^68^^,^^125^ Similarly, genera such as *Parabacteroides*, *Barnesiella*, *Odoribacter*, *Blautia*, *Eggerthella*, and *Ruminococcus* include strains with divergent metabolic capacities, making broad functional generalizations potentially misleading.^126^^,^^127^ This strain dependence extends to microbial metabolites. The same genus may support neurodevelopment under one ecological configuration by promoting SCFA production, vitamin synthesis, or anti-inflammatory signaling, yet behave differently in a dysbiotic environment characterized by succinate accumulation, inflammatory metabolites, or excessive mucin degradation. For this reason, functional output and ecological context are more informative than taxonomy alone.

### 3.4. Integrative interpretation for the rest of the review

The key message for the sections that follow is that neurodevelopmentally relevant microbial programming involves a recurring set of biological processes: establishment of early commensal-rich ecosystems, production of SCFAs and other neuroactive metabolites, maintenance of epithelial and BBB integrity, calibration of immune responses, and limitation of inflammatory spillover from dysbiotic states.^11^^,^^15^^,^^91^ The importance of specific maternal niches, including gut, vagina, milk, skin, and oral communities, lies in how they deliver or shape these functions across pregnancy, birth, and infancy. This framework also clarifies why *Bacteroides*, *Bifidobacterium*, and *Lactobacillus* should remain central throughout the review: they represent key anchors of fecal, milk-associated, and vaginal transmission pathways that converge on early immune and neurodevelopmental programming. Conversely, dysbiosis-associated enrichment of inflammatory pathobionts should be interpreted as a modifier of developmental risk rather than as deterministic proof of causation. The following sections therefore focus on routes of transfer and timing of exposure, while referring back to these core microbial mechanisms.

## 4. Routes of parental microbial transmission across early-life

Building on the mechanistic framework outlined above, early-life microbial programming occurs through a series of temporally ordered transmission routes that connect parental microbiomes with offspring neurodevelopment. While Section 3 established the key microbial taxa, metabolites, and signaling pathways involved in neurodevelopment, the following sections examine how these signals are transferred across developmental windows, spanning gestation, birth, and early postnatal life. This structure integrates microbial composition with biological timing, clarifying how distinct maternal niches act as sequential gateways that shape the developing MGBA. Together, these transmission routes form a continuous, multi-stage process through which maternal (and, indirectly, paternal) microbial signals influence offspring brain development.

### 4.1. Placental interface as a microbial gatekeeper

**Figure 3.**
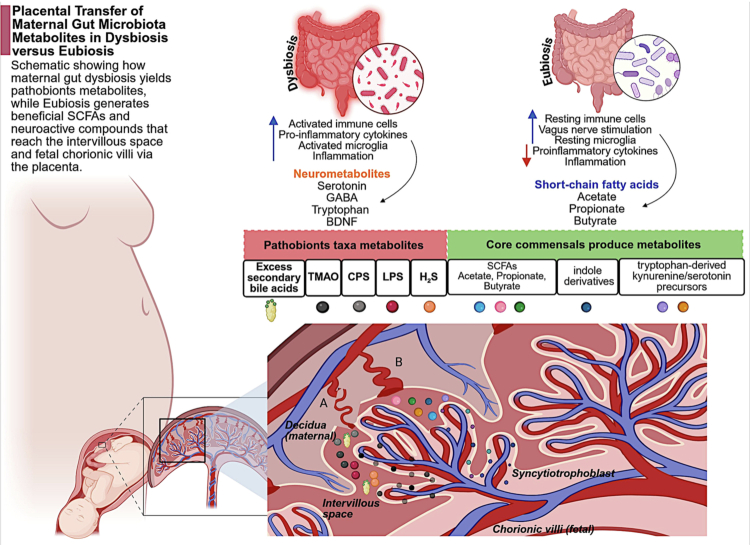
Placental transfer of maternal gut microbiota–derived metabolites under eubiosis and dysbiosis. Schematic illustration of how maternal gut microbial states influence the transfer of metabolites across the placenta. Under eubiosis, commensal-derived metabolites, including SCFAs, indole derivatives, and tryptophan-related metabolites, promote immune homeostasis and support fetal neurodevelopment. In contrast, dysbiosis is associated with increased production of pathobiont-derived metabolites, including lipopolysaccharide (LPS), trimethylamine-*N*-oxide (TMAO), hydrogen sulfide (H₂S), capsular polysaccharides (CPS), and secondary bile acids, which may enhance placental inflammation, immune activation, and microglial priming. These metabolites reach the intervillous space and can be transported across the syncytiotrophoblast to the fetal chorionic villi. Abbreviations: SCFAs, short-chain fatty acids; LPS, lipopolysaccharide; TMAO, trimethylamine-*N*-oxide; CPS, capsular polysaccharides; H₂S, hydrogen sulfide. (Created with BioRender).

Current evidence does not support the presence of a consistent, viable placental microbiome. Early reports identifying bacterial DNA within placental tissues have largely been attributed to contamination and low-biomass artifacts, and subsequent metagenomic analyzes have failed to demonstrate reproducible colonization by live microbes.^14^ Instead, the placenta functions as a highly selective biological interface that transmits microbial metabolites, immune mediators, and host-derived signals from the maternal circulation to the developing fetus. This selective transfer is primarily mediated by the syncytiotrophoblast, a continuous epithelial layer that regulates molecular exchange through membrane transporters, receptor-mediated endocytosis, and transcytosis.^128^ Through this system, microbiota-derived metabolites can reach the fetal compartment without direct microbial translocation. For example, SCFAs, key products of maternal gut microbial fermentation, are detectable in cord blood and correlate with maternal circulating levels, indicating placental transfer.^129^ These metabolites have been implicated in fetal neurodevelopment, particularly in experimental models, through roles in microglial maturation, BBB integrity, and epigenetic regulation.^92^^,^^130^ In parallel, placental tryptophan metabolism represents a critical interface between maternal microbiota and fetal brain development. The enzyme indoleamine-2,3-dioxygenase (IDO) converts maternal tryptophan into kynurenine-pathway metabolites that cross the placenta and influence neurodevelopmental processes.^131^ Although direct human evidence linking specific microbiota-derived tryptophan metabolites to neurodevelopment remains limited, disruptions in placental serotonin and tryptophan signaling have been associated with altered fetal brain development.^132^ Microbial indole derivatives, such as indole-3-propionic acid, may further contribute to placental homeostasis and fetal protection, although their precise role requires further clarification.^133^ A schematic overview of placental transfer of commensal- and dysbiosis-associated maternal microbial metabolites is shown in Figure 3.

Bile acids constitute another major class of microbiota-associated metabolites that traverse the placenta. Under physiological conditions, tightly regulated maternal–fetal gradients maintain controlled fetal exposure. However, in pathological states such as intrahepatic cholestasis of pregnancy, dysregulated bile acid transport leads to fetal accumulation and has been associated with altered neuronal differentiation and stress responses.^134^ Experimental models demonstrate that normalization of bile acid transport using ursodeoxycholic acid can mitigate adverse metabolic and neurodevelopmental outcomes in offspring,^135^ highlighting the functional significance of this pathway. In addition to metabolic signaling, maternal immune activation represents a critical mechanism linking the maternal microbiome to fetal brain development. Elevated maternal cytokines, particularly interleukin-6 and interleukin-17A, can signal across the placenta and disrupt cortical development, leading to long-term behavioral alterations in offspring in animal models.^136^^,^^137^ These findings support a model in which maternal microbiome-driven immune responses influence fetal neurodevelopment indirectly through inflammatory signaling pathways. Anatomically, maternal blood carrying microbial metabolites and immune mediators enters the intervillous space, where it interfaces with the syncytiotrophoblast. Beneath this layer, fetal capillaries within the chorionic villi receive selectively transported molecules. Immune cells within the decidua, including macrophages and T cells, further modulate this environment by responding to microbial and inflammatory signals. This spatial organization enables differential transfer of molecular signals: commensal-derived metabolites such as SCFAs and indoles generally support neurodevelopmental processes, whereas pathobiont-associated molecules such as lipopolysaccharide and secondary bile acids promote inflammatory signaling that may disrupt fetal brain development. Collectively, the placenta functions not as a passive barrier but as an active regulatory interface that integrates maternal microbial, metabolic, and immune signals. Through selective transfer mechanisms, it shapes fetal exposure to neuroactive molecules, thereby establishing an early foundation for MGBA programming prior to birth.

### 4.2. Vaginal delivery: a gatekeeper of early microbial and neurodevelopmental signaling

Mode of delivery represents a critical postnatal gateway linking the maternal microbiome to early-life microbial colonization and neurodevelopment. During vaginal birth, the neonate is exposed to maternal vaginal and fecal microbiota, including key taxa such as *Lactobacillus*, *Bacteroides*, and *Bifidobacterium*, which initiate gut colonization and early immune programming.^16^^,^^100^^,^^106^^,^^138^ In contrast, Cesarean delivery limits exposure to these maternal reservoirs and favors colonization by skin- and environmental-associated microbes, including *Staphylococcus* and *Streptococcus*.^107^ These differences are most pronounced during the first months of life, a period characterized by rapid synaptogenesis, immune maturation, and MGBA development.^139^^,^^140^ Vaginally delivered infants typically acquire maternal strains that promote ecological succession and metabolic functionality of the gut microbiome, whereas cesarean-born infants often exhibit delayed colonization by key taxa such as *Bacteroides* and *Bifidobacterium*. These genera are important for SCFA production, tryptophan metabolism, and immune modulation, processes implicated in microglial maturation, neurotransmitter balance, and early neural circuit formation.^11^^,^^108^

Epidemiological studies have associated cesarean delivery with increased risks of neurodevelopmental disorders, including autism spectrum disorder, attention-deficit/hyperactivity disorder, language delay, and emotional dysregulation.^141^ However, these associations should be interpreted cautiously, as they may reflect confounding factors such as maternal health status, antibiotic exposure, or obstetric complications rather than delivery mode alone. Thus, while altered microbial seeding is a plausible mechanism, causality remains unresolved. Experimental evidence supports a mechanistic role of delivery-associated signals. Natural birth has been linked to regulation of hippocampal mitochondrial function and neuronal differentiation,^142^ while early microbial exposure influences cytokine, metabolic, and vagal signaling pathways relevant to stress regulation and cognitive development.^143^ Importantly, emerging strategies aim to mitigate cesarean-associated dysbiosis. Vaginal microbiota transfer has been shown to partially restore infant gut microbial composition toward vaginal delivery profiles and may improve early developmental metrics, although current evidence remains preliminary.^144^ These approaches highlight a translational opportunity but require further validation regarding safety and long-term neurodevelopmental outcomes. Overall, vaginal delivery acts as a foundational microbial and neurodevelopmental event, although its long-term impact is modulated by subsequent exposures such as breastfeeding, environment, and antibiotic use.

### 4.3. Milk microbiome as a determinant of postnatal neurodevelopmental trajectories

**Figure 4.**
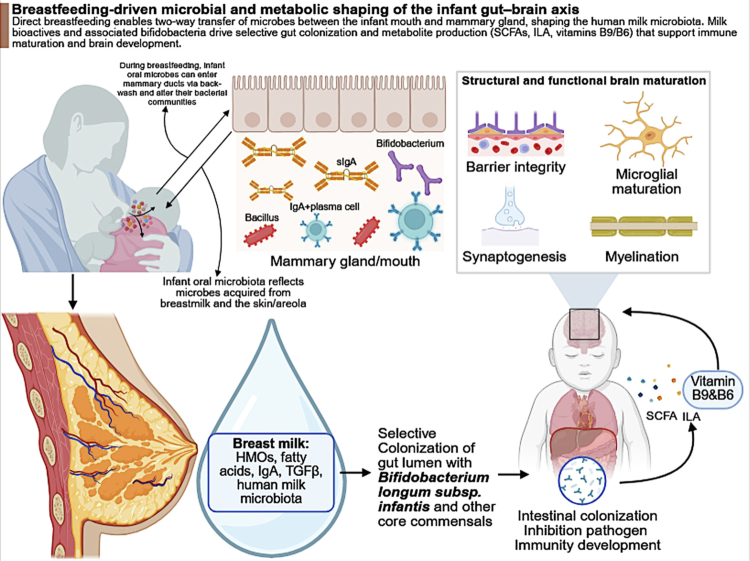
Breastfeeding-driven microbial and metabolic shaping of the infant gut–brain axis. Schematic overview of how breastfeeding supports bidirectional microbial exchange between the maternal mammary gland and the infant, contributing to early gut colonization and neurodevelopment. Breast milk delivers human milk oligosaccharides (HMOs), immunoglobulins (e.g., sIgA), fatty acids, cytokines (e.g., TGF-*β*), and microbial taxa, promoting selective colonization by *Bifidobacterium longum* subsp. *infantis* and other commensals. Microbial metabolism generates SCFAs, indole-3-lactic acid (ILA), and vitamins (e.g., B9 and B6), which contribute to intestinal barrier integrity, immune maturation, and brain development through processes including synaptogenesis, myelination, microglial maturation, and BBB stabilization. Abbreviations: HMO, human milk oligosaccharides; sIgA, secretory immunoglobulin A; TGF-*β*, transforming growth factor beta; SCFAs, short-chain fatty acids; ILA, indole-3-lactic acid. (Created with BioRender).

Following birth, human milk represents a central route of continued maternal microbial and biochemical influence. This postnatal pathway, including microbial transfer, milk bioactives, and downstream effects on infant gut and brain development, is illustrated in Figure 4. Beyond nutrition, breast milk delivers microbes, human milk oligosaccharides (HMOs), immune factors, and neuroactive compounds that collectively shape infant gut colonization and neurodevelopment.^41^^,^^145^^,^^146^ The milk microbiome contains diverse taxa, including *Staphylococcus*, *Streptococcus*, *Propionibacterium*, *Bacteroides*, *Blautia*, *Clostridium*, *Roseburia*, and *Ruminococcus*, with evidence of strain-level overlap between maternal milk and infant gut microbiota.^68^^,^^99^ This supports ongoing vertical microbial transmission during lactation, although distinguishing direct microbial transfer from substrate-driven selection remains challenging.

HMOs play a central role by selectively enriching *Bifidobacterium longum* subsp. *infantis* and related taxa, promoting gut barrier integrity and immune maturation.^147^ Additional components such as secretory IgA, lactoferrin, and antimicrobial peptides regulate microbial colonization, while fatty acids and neurotrophic factors contribute directly to brain development.^148^^,^^149^ Neurodevelopmental associations are supported by both observational and mechanistic studies. HMOs such as 2′-fucosyllactose and *N*-acetylneuraminic acid have been linked to improved synaptogenesis, motor skills, and white matter development.^146^ Exclusive breastfeeding has also been associated with improved cognitive and behavioral outcomes, although these effects likely reflect combined biological and environmental influences.^150^ More direct microbiome-mediated evidence suggests that maternal milk microbial composition may influence infant neurodevelopment via modulation of the infant gut microbiome and SCFA production.^151^ This supports a model in which non-gut maternal microbiomes contribute indirectly to neurodevelopment by shaping the infant gut ecosystem. However, current evidence remains largely observational, and milk composition is influenced by multiple factors including maternal diet, metabolic status, delivery mode, and antibiotic exposure.^67^^,^^69^ Therefore, causal interpretations should be made cautiously. Overall, the milk microbiome represents a sustained postnatal extension of maternal microbial programming, integrating microbial transfer, metabolic signaling, and immune regulation to shape early neurodevelopment.

### 4.4. Early environmental and tactile routes of microbial–neurodevelopmental programming

**Figure 5.**
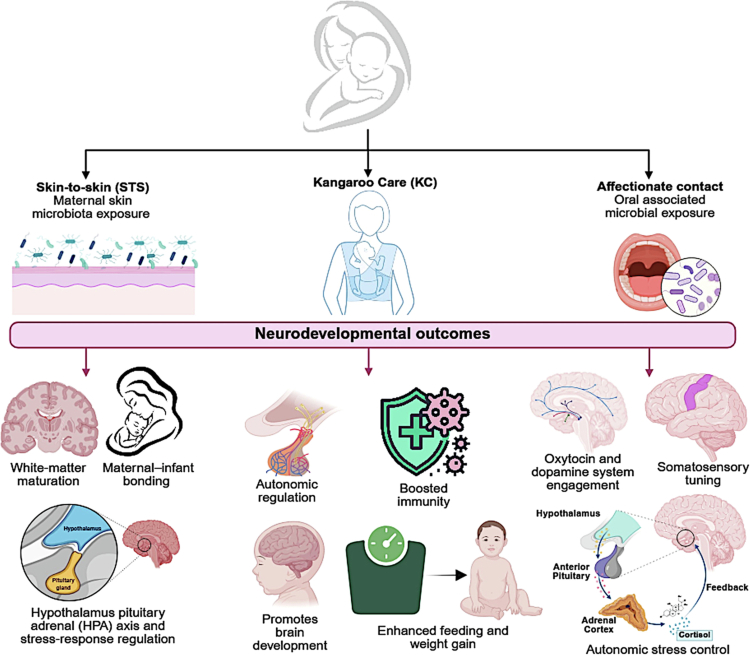
Early postnatal tactile and close-contact pathways linking maternal care to infant microbial and neurodevelopmental programming. Schematic representation of early postnatal maternal–infant interactions that influence microbial exposure and neurodevelopment. Skin-to-skin (STS) contact facilitates transfer of maternal skin-associated microbiota. Kangaroo care (KC), a structured form of prolonged STS, primarily enhances autonomic regulation and stress resilience. Affectionate contact and close proximity contribute to exposure to maternal oral and skin microbiota. These interactions are associated with improved neurodevelopmental outcomes, including white matter maturation, hypothalamic–pituitary–adrenal (HPA) axis regulation, immune development, feeding behavior, and socioemotional and sensory processing. Abbreviations: STS, skin-to-skin; KC, kangaroo care; HPA, hypothalamic–pituitary–adrenal. (Created with BioRender).

Beyond delivery and breastfeeding, early-life environmental and tactile exposures provide additional pathways linking maternal microbiota and neurodevelopment. These overlapping tactile and close-contact pathways are summarized schematically in Figure 5. These include skin-to-skin contact, kangaroo care, affectionate touch, and environmental microbial exposure. Skin-to-skin contact facilitates exposure to maternal skin-associated microbiota and has been associated with changes in infant oral and gut microbial composition, including enrichment of taxa such as *Gemella*, *Aggregatibacter*, and members of the *Lachnospiraceae* family.^152^ However, whether these changes represent stable colonization or transient exposure remains unclear.

Kangaroo care, a structured form of prolonged skin-to-skin contact, has well-established neurodevelopmental benefits, including improved autonomic regulation, stress resilience, and behavioral organization in preterm infants.^153^^,^^154^ Early-life stressors such as maternal deprivation have also been associated with long-term alterations in inhibitory interneuron number and morphology in limbic brain regions.^155^^,^^156^These effects are likely mediated primarily through neuroendocrine and autonomic pathways, with microbial contributions remaining secondary and less clearly defined. Affectionate caregiver touch, including holding and physical interaction, influences HPA axis activity, neural synchrony, and socioemotional development.^157^^,^^158^ These interactions may also contribute to microbial exchange, although their primary impact appears neuroregulatory rather than microbiological. Environmental exposures further shape early microbial development. Infants raised in farm environments exhibit distinct gut microbiota enriched in taxa such as *Clostridiaceae*, *Akkermansia*, and *Blautia*, which are associated with SCFA production and immune conditioning.^159^ While these findings suggest broader ecological influences on neurodevelopment, direct mechanistic links remain limited. Overall, these routes act as modulatory pathways that reinforce or reshape microbial and neurodevelopmental trajectories established earlier, rather than serving as primary drivers.

### 4.5. Integrative perspective

Maternal microbial programming occurs through a sequence of interconnected transmission routes across developmental time. During pregnancy, the placenta mediates transfer of microbial metabolites and immune signals. At birth, vaginal delivery seeds the neonatal microbiome. During infancy, breast milk sustains microbial and metabolic signaling, while environmental and tactile exposures further modulate these processes. Together, these pathways form a temporally coordinated framework through which maternal multi-niche microbiomes shape the infant MGBA. Disruptions at different stages, including cesarean delivery, impaired breastfeeding, antibiotic exposure, or maternal dysbiosis, may therefore have distinct but overlapping consequences for neurodevelopment.

## 5. Maternal diet, probiotics, and microbiome modulation of offspring neurodevelopment

**Figure 6.**
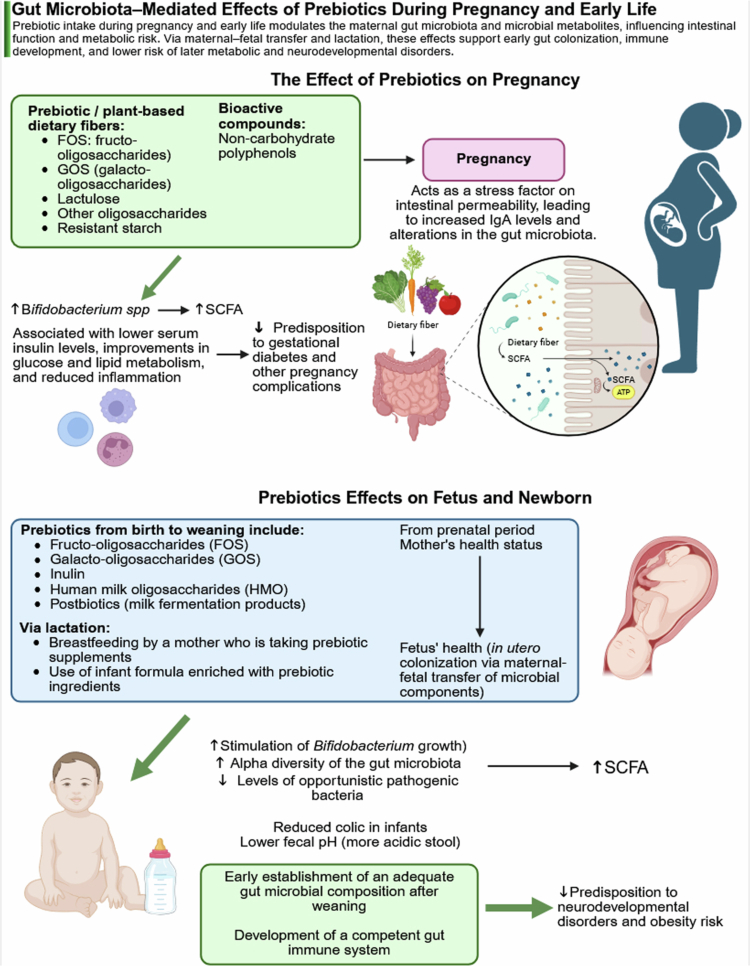
Diet–microbiota interactions during pregnancy and early-life. Schematic overview of how maternal dietary components, particularly prebiotic fibers and polyphenols, modulate gut microbiota composition and metabolic activity during pregnancy and early life. Fermentation of dietary fibers such as fructooligosaccharides (FOS), galactooligosaccharides (GOS), and resistant starch promotes growth of beneficial taxa (e.g., *Bifidobacterium*) and production of SCFAs, which influence maternal metabolic status, placental signaling, and early-life gut colonization. These effects extend to the infant through prenatal exposure and lactation, supporting immune maturation and potentially reducing the risk of later metabolic and neurodevelopmental disorders. Abbreviations: FOS, fructooligosaccharides; GOS, galactooligosaccharides; SCFAs, short-chain fatty acids. (Created with BioRender).

Maternal diet represents one of the most powerful and modifiable regulators of the MGBA during pregnancy and lactation. Dietary inputs shape maternal microbial composition and metabolic output, which in turn influence fetal and infant neurodevelopment through microbial metabolites, immune modulation, and vertical transmission pathways.^160^ Rather than acting through isolated mechanisms, dietary components converge on shared pathways, particularly SCFA production, inflammatory signaling, and microbial inheritance, linking maternal nutrition to offspring brain development.^161-164^ Representative human and experimental studies evaluating maternal microbiome-targeted interventions and offspring outcomes are summarized in Table 1. Selected diet–microbiome interactions relevant to pregnancy and early-life programming, particularly those involving prebiotic and fiber-associated pathways, are summarized in Figure 6.

**Table 1.** Selected studies on maternal microbiome-targeted interventions and offspring outcomes.

Sample size	Intervention	Duration	Outcome	Key finding	Reference
200 pregnant women	Multispecies probiotic vs placebo	3rd trimester - 6 months postpartum	Infant gut colonization, maternal inflammation	Increased Bifidobacterium and Lactobacillus abundance; decreased inflammation; safe and beneficial for maternal–infant microbiota balance	[109]
150 pregnant women	Multispecies probiotic supplement	During pregnancy to infant follow up	Neurodevelopmental outcomes	Probiotic intake improved infant cognitive and social behavior, with no adverse effects reported	[165]
250 mother infant pairs	Observational (maternal microbiome diversity)	Pregnancy to 2 years postpartum	Infant cognition and motor development	Higher Bacteroides and Faecalibacterium abundance associated with better cognitive and fine motor outcome	[166]
120 couples	Mediterranean style diet adherence	Preconception to late pregnancy	Maternal microbiome composition, infant metabolic profile	Diet increased SCFA-producing taxa, leading to improved infant metabolic resilience	[167]
160 pregnant women	Maternal microbial modulation during gestation and lactation	Pregnancy through lactation	Infant growth and microbiome establishment	Maternal microbes were transmitted to offspring, enhancing gut colonization and growth	[11]
Animal + human ex vivo validation	Maternal gut microbiota modulation	Gestation	Fetal stem cell function, neurodevelopment	Maternal microbiota metabolites regulate offspring neuronal and intestinal stem cells via mTOR signaling	[168]
Animal model	High fiber diet	Maternal obesity model	Offspring cognition, microbiome metabolites	High-fiber diet restored SCFA levels and reduced neurobehavioral deficits in offspring	[169]

### 5.1. Dietary fiber and SCFA-mediated neurodevelopmental programming

Dietary fiber is a central driver of maternal gut microbial metabolism. Fermentation of fiber by commensal bacteria generates SCFAs, including acetate, propionate, and butyrate, which act as key signaling molecules within the MGBA. These metabolites can cross the placenta and have been detected in fetal circulation, where they influence microglial maturation, synaptic development, and epigenetic regulation.^162^^,^^170-172^ These effects extend to structural neuronal development, as microbial and metabolic perturbations have been shown to alter dendritic morphology and cortical circuit organization.^173^^,^^174^ Clinical and preclinical studies consistently show that higher maternal fiber intake is associated with enrichment of SCFA-producing taxa such as *Faecalibacterium*, *Roseburia*, and *Lachnospira*, alongside reduced abundance of pro-inflammatory genera including *Collinsella.*^175^ In contrast, low-fiber diets promote dysbiosis and reduce circulating SCFAs, limiting fetal exposure to neuroactive metabolites. Experimental models further demonstrate that maternal fiber deficiency impairs offspring synaptic plasticity, alters hippocampal signaling pathways, and leads to behavioral deficits, which can be partially rescued by butyrate supplementation.^170^ Beyond gestation, lactation represents an additional window of microbiome-mediated programming. Maternal fiber intake influences milk composition and microbial transfer, shaping early gut colonization and immune maturation in the offspring.^176^ Collectively, these findings position dietary fiber as a key upstream regulator of SCFA-dependent neurodevelopmental pathways.

### 5.2. Polyphenols and microbiome-derived neuroactive metabolites

Polyphenols interact bidirectionally with the gut microbiota, acting both as substrates for microbial metabolism and as modulators of microbial community structure. Microbial conversion of polyphenols generates bioactive metabolites, including urolithins and phenolic acids, which exhibit anti-inflammatory and neuroprotective properties.^177^^,^^178^ Maternal polyphenol intake has been associated with enrichment of beneficial taxa such as *Akkermansia* and *Blautia*, alongside reductions in lipopolysaccharide-associated bacteria.^179^^,^^180^ These shifts promote a more anti-inflammatory microbial environment, which may indirectly support fetal brain development through reduced systemic inflammation and improved barrier integrity. Evidence from human studies indicates that microbial polyphenol metabolites can be transferred via breast milk, linking maternal diet to infant gut colonization. For example, urolithins derived from maternal intake have been detected in both breast milk and infant biological samples, accompanied by increased abundance of butyrate-producing taxa in the infant gut.^181^ While direct causal links to neurodevelopment remain limited, these findings support a microbiome-mediated pathway through which maternal polyphenol intake may influence early-life programming.

### 5.3. High-fat diet, dysbiosis, and neuroinflammatory risk

**Figure 7.**
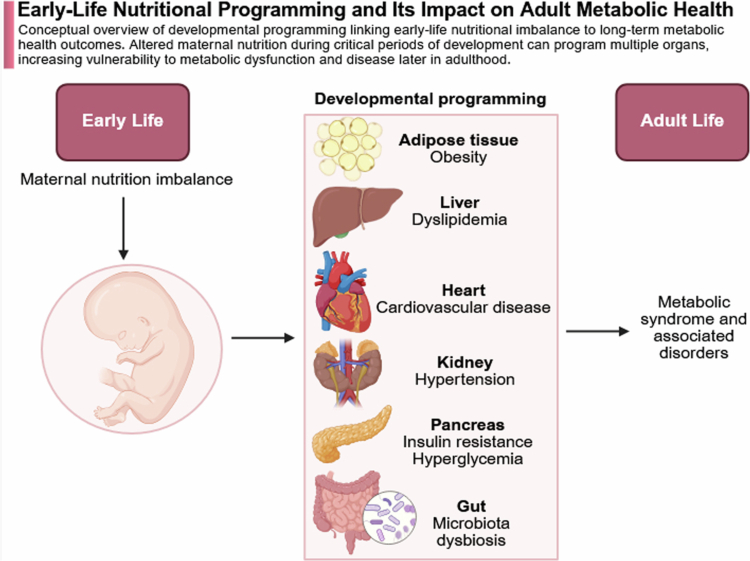
Early-life nutritional programming and its impact on adult metabolic health. Conceptual model illustrating how early-life nutritional imbalance influences long-term metabolic health through developmental programming. Altered maternal nutrition during critical windows of fetal development can affect multiple organ systems, including adipose tissue, liver, heart, pancreas, kidney, and gut microbiota. These changes may predispose individuals to metabolic syndrome, insulin resistance, cardiovascular disease, and microbiome dysbiosis in adulthood. (Created with BioRender).

In contrast to fiber- and polyphenol-rich diets, maternal high-fat diet (HFD) induces microbial dysbiosis characterized by reduced diversity, depletion of SCFA-producing bacteria, and enrichment of pro-inflammatory taxa.^182^^,^^183^ These microbial alterations are associated with increased intestinal permeability and elevated circulating lipopolysaccharide, promoting systemic inflammation. Maternal inflammation represents a key mechanistic link between HFD-induced dysbiosis and altered neurodevelopment. Experimental studies demonstrate that maternal HFD exposure disrupts fetal microglial maturation, alters glutamatergic signaling, and induces behavioral abnormalities in offspring, including anxiety-like behavior and cognitive deficits.^184^ These effects are partly mediated by altered microbial metabolite profiles, including reduced SCFAs and dysregulated tryptophan metabolism. Importantly, these microbiome alterations are vertically transmitted, with offspring of HFD-fed mothers exhibiting persistent dysbiosis despite postnatal dietary normalization.^185^^,^^186^ Together, these findings highlight maternal HFD as a key disruptor of microbiome-dependent neurodevelopmental programming. The broader concept of early-life nutritional programming and its downstream metabolic consequences is illustrated in Figure 7.

### 5.4. Probiotics and targeted microbiome modulation

Probiotic supplementation has emerged as a potential strategy to modulate the maternal microbiome and influence offspring neurodevelopment. Most evidence focuses on *Bifidobacterium* and *Lactobacillus* species, which are associated with SCFA production, immune regulation, and early-life gut colonization.^111^ Clinical studies indicate that maternal probiotic supplementation can increase beneficial taxa and reduce pro-inflammatory bacteria in both mother and infant.^109^ These microbial changes are associated with enhanced gut barrier function, reduced inflammation, and improved early microbial colonization patterns. In some studies, probiotic exposure has also been linked to improved neurobehavioral outcomes, although evidence remains limited and strain-specific.^165^ Mechanistically, probiotics may act through multiple pathways, including SCFA production, modulation of cytokine signaling, and vertical microbial transmission via breast milk. Notably, probiotic-induced changes in milk microbiota and metabolites have been associated with altered infant gut composition and early cognitive markers.^187^ However, heterogeneity across studies highlights the need for standardized interventions and mechanistic validation.

### 5.5. Integrative perspective

Maternal diet and microbiome-targeted interventions converge on shared biological pathways that regulate offspring neurodevelopment. Diets rich in fiber and polyphenols promote SCFA production, microbial diversity, and anti-inflammatory signaling, supporting neurodevelopmental resilience. In contrast, high-fat diets disrupt these pathways, promoting dysbiosis and neuroinflammatory risk. Probiotic interventions offer a targeted approach to partially restore microbial balance, although their effects depend on strain, timing, and host context. Overall, maternal nutrition shapes offspring neurodevelopment not only through direct nutrient supply but also through microbiome-mediated signaling networks. These findings reinforce the maternal microbiome as a central and modifiable interface linking environment, metabolism, and brain development across generations.

## 6. Paternal microbiome: an emerging contributor to neurodevelopmental programming

### 6.1. Paternal microbiome and the gut–germline axis

**Figure 8.**
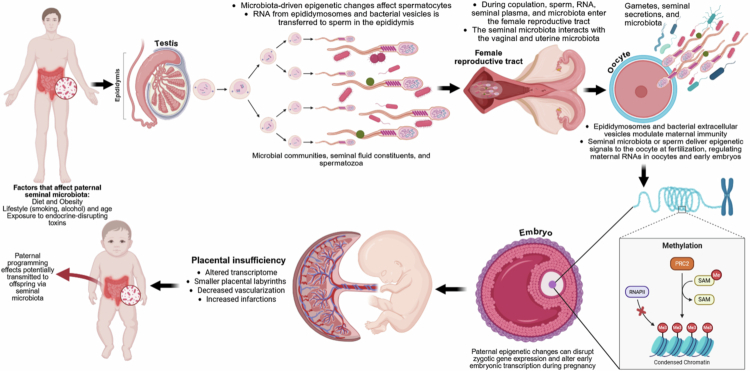
Paternal microbiome and germline-mediated effects on offspring development. Schematic representation of paternal microbiome–mediated pathways influencing offspring development. Paternal factors such as diet, lifestyle, and environmental exposures shape gut and seminal microbiota, which can influence spermatogenesis and sperm molecular content, including RNA cargo and epigenetic marks. During fertilization, sperm, seminal plasma, and associated microbial signals interact with the maternal reproductive tract, contributing to immune modulation and early embryonic programming. These processes may influence placental development, embryonic gene expression, and offspring physiological and neurodevelopmental outcomes. Abbreviations: RNA, ribonucleic acid. (Created with BioRender).

Paternal contributions to offspring development extend beyond genetic inheritance and increasingly include microbiome-mediated mechanisms. A schematic overview of paternal microbiome- and germline-mediated pathways relevant to offspring development is presented in Figure 8. Within the developmental origins of health and disease framework, the paternal microbiome, particularly the gut microbiota, acts as a regulator of germline signaling prior to conception.^12^^,^^188^ Experimental evidence supports the existence of a gut–germline axis, whereby alterations in the paternal gut microbiome influence sperm molecular composition. Disruption of the paternal microbiota has been shown to modify sperm small non-coding RNA profiles, including piRNAs, which play a key role in early embryonic gene regulation.^188^ These microbiome-driven changes are associated with downstream effects in offspring, including altered metabolism, gut development, and behavior, suggesting that paternal microbial status may contribute to early-life programming. Importantly, these findings position the paternal microbiome as a mediator between environmental exposures, such as diet, stress, and antibiotics, and germline signaling, expanding the concept of intergenerational inheritance beyond maternal pathways.

### 6.2. Seminal microbiome and reproductive tract interactions

In addition to gut-mediated effects, the seminal microbiome represents a direct route through which paternal microbial signals may influence early development. Contrary to earlier assumptions, semen is not sterile but contains a diverse microbial community, including commensal and opportunistic taxa that coexist with spermatozoa and bioactive molecules.^12^^,^^189^ These seminal microbial communities are transferred to the female reproductive tract at conception, where they interact with maternal vaginal and uterine microbiota. This interaction may influence immune tolerance, inflammatory balance, and implantation processes, thereby indirectly shaping embryonic development. Conceptual models, such as the semino-vaginal microbiome framework, propose that microbial signals from both parents converge at the maternal–fetal interface to influence early developmental trajectories.^12^ Although the composition and function of the seminal microbiome remain incompletely characterized, emerging evidence suggests associations with sperm quality, oxidative stress, and reproductive outcomes.^190^ However, mechanistic links to offspring neurodevelopment remain largely indirect and require further investigation.

### 6.3. Epigenetic inheritance: mechanisms and controversies

**Figure 9.**
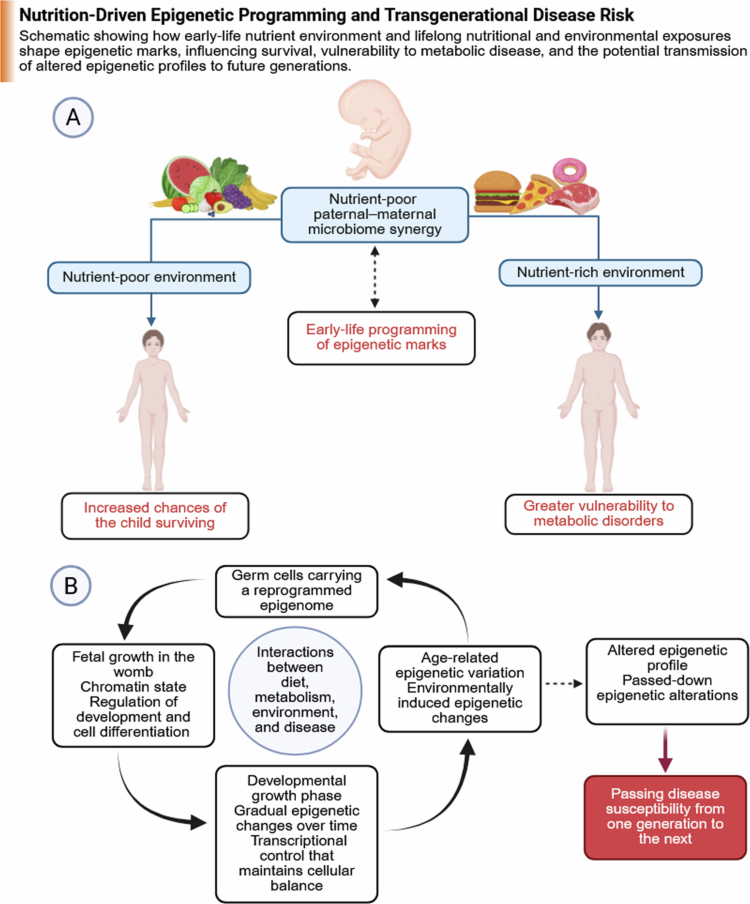
Nutrition-driven epigenetic programming and transgenerational disease risk. Schematic illustrating how early-life nutritional and environmental exposures influence epigenetic regulation and long-term disease susceptibility across generations. Panel (A) depicts how nutrient-rich versus nutrient-poor environments shape early-life programming and developmental outcomes. Panel (B) shows how epigenetic mechanisms, including DNA methylation and histone modifications, regulate gene expression during fetal development and throughout life. These epigenetic changes may persist, contributing to metabolic disease risk and potentially being transmitted across generations. Abbreviations: DNA, deoxyribonucleic acid. (Created with BioRender).

Epigenetic inheritance represents a key pathway through which paternal environmental and microbial signals may influence offspring development. The broader relationship between developmental epigenetic programming and transgenerational disease susceptibility is illustrated in Figure 9. Sperm carries multiple layers of epigenetic information, including DNA methylation, retained histone modifications, and small non-coding RNAs, all of which can regulate early embryonic gene expression.^191^^,^^192^ Among these mechanisms, small non-coding RNAs are particularly sensitive to environmental and microbiome-related influences. Alterations in sperm RNA content have been shown to affect early embryonic transcriptional programs, including pathways related to metabolism, stress response, and neurodevelopment.^188^ However, the extent to which paternal epigenetic modifications are transmitted across generations remains a subject of ongoing debate. During early embryogenesis, extensive epigenetic reprogramming occurs, which erases most parental epigenetic marks. While certain regions, such as imprinted genes, escape this reprogramming, it remains unclear how consistently environmentally induced epigenetic changes persist and influence long-term outcomes.^193^ Similarly, evidence for histone-based inheritance is emerging but remains limited. For example, paternal obesity has been associated with altered histone modifications in sperm that may influence early embryonic gene expression, although causality and persistence are not fully established.^194^ Overall, while epigenetic mechanisms provide a plausible link between paternal microbiome status and offspring development, current evidence remains incomplete, and further research is required to resolve these uncertainties.

### 6.4. Integration of paternal and maternal microbiome contributions

Rather than acting independently, paternal and maternal microbiomes likely function as an integrated system during early development. Microbial signals from both parents converge during critical windows, including fertilization, implantation, and early gestation, shaping the embryonic environment through combined metabolic, immune, and epigenetic influences.^12^^,^^195^ Paternal contributions, mediated through sperm epigenetics and seminal microbiota, interact with maternal microbiome-derived signals that dominate during pregnancy and early infancy. This convergence may influence placental development, immune tolerance, and early microbial colonization, ultimately impacting neurodevelopmental trajectories. Although human evidence remains limited, this integrated parental framework provides a more comprehensive model of intergenerational programming and aligns with emerging systems-level approaches to microbiome research.

### 6.5. Integrative perspective

The paternal microbiome represents an emerging but still underexplored component of microbiome–gut–brain axis research. Evidence from experimental and observational studies suggests that paternal microbial status influences offspring development through interconnected pathways involving the gut microbiome, seminal microbiota, and epigenetic signaling. However, compared with maternal pathways, paternal mechanisms remain less well defined, and current evidence is often indirect or derived from animal models. Future studies should aim to clarify the relative contribution of paternal microbiome pathways, their interaction with maternal systems, and their relevance to human neurodevelopment.

## 7. Integrative multi-omics and systems biology approaches

### 7.1. Conceptual framework: linking microbiome, metabolism, and neurodevelopment

Advances in multi-omics approaches have enabled a more integrated understanding of how the microbiome contributes to neurodevelopment through interconnected metabolic, immune, and epigenetic pathways. Rather than acting in isolation, the maternal and early-life microbiome interacts with host systems across multiple biological layers, including microbial composition (metagenomics), metabolite production (metabolomics), and host gene regulation (epigenomics).^196^^,^^197^ In the context of the MGBA, these layers converge to influence key developmental processes such as microglial maturation, synaptic formation, and BBB integrity. Microbiota-derived metabolites, including SCFAs, tryptophan derivatives, and bile acids, act as central mediators linking gut microbial activity to brain development.^92^^,^^108^ Integrating multi-omics data therefore provides a systems-level framework to understand how early-life microbial exposures shape neurodevelopmental trajectories.

### 7.2. Multi-omics evidence linking the microbiome to neurodevelopment

Emerging multi-omics studies in pediatric populations demonstrate that combined microbiome–metabolome analyzes provide deeper insight into neurodevelopment than single-layer approaches. For example, integrative analyzes combining metagenomics and metabolomics have identified coordinated alterations in microbial taxa and neuroactive metabolites associated with neurodevelopmental disorders, including disruptions in SCFA pathways and neurotransmitter-related metabolism.^198^ Importantly, these studies highlight that microbiome-derived metabolites with BBB permeability may serve as functional mediators of gut–brain communication. For instance, alterations in microbial pathways involved in tryptophan metabolism and SCFA production have been linked to changes in brain connectivity, immune activation, and behavioral outcomes.^166^ These findings support a model in which microbial function, rather than taxonomy alone, is a key determinant of neurodevelopmental outcomes.

### 7.3. Microbiome–brain integration: toward mechanistic models

Integrating microbiome data with neurobiological readouts, including neuroimaging and behavioral assessments, represents a critical step toward mechanistic understanding. Studies combining microbial profiling with neurodevelopmental outcomes demonstrate associations between early-life microbiome composition and brain structure, connectivity, and cognitive performance.^199^ Machine learning approaches have further enabled the identification of microbiome-based signatures associated with neurodevelopmental phenotypes, although current models remain limited by cohort size and heterogeneity.^200^ Despite these limitations, such integrative frameworks support the concept that microbiome-derived signals contribute to neurodevelopment through coordinated effects on metabolism, immune signaling, and neural circuit formation.

### 7.4. Challenges and future directions

Despite its potential, multi-omics integration in microbiome research faces several challenges. These include variability across cohorts, differences in sampling time points, and difficulty distinguishing causal relationships from associations. Additionally, the dynamic nature of the microbiome during pregnancy and early-life complicates longitudinal interpretation.^200^ Importantly, moving from descriptive associations to causal mechanisms will be essential for translating microbiome research into clinical applications targeting neurodevelopment. Future studies should prioritize longitudinal mother–infant cohort designs that capture dynamic microbiome changes across developmental windows, alongside integrative analyzes combining microbial, metabolomic, and neurodevelopmental data. Clear distinction between findings derived from human studies and animal models will be essential to improve translational relevance. In addition, functional validation of microbiome-derived metabolites is needed to establish causal mechanisms linking microbial activity to neurodevelopmental outcomes.

## 8. Conclusion, translational implications, and future directions

### 8.1. Integrated model of parental microbiome programming of early-life neurodevelopment

Current evidence supports a model in which early-life neurodevelopment is shaped by a dynamic parental microbial network acting across preconception, pregnancy, birth, and infancy. Within this framework, the maternal microbiome remains the dominant source of direct early microbial and metabolite exposure, but paternal contributions are increasingly recognized as biologically relevant modifiers of developmental programming. Across the maternal gut, vaginal, milk, oral, and skin niches, microbial communities influence offspring development through partially overlapping mechanisms that include metabolite transfer, immune modulation, vertical microbial transmission, and regulation of early gut colonization. These processes converge on the microbiome–gut–brain axis, where microbial products and host responses shape intestinal barrier maturation, microglial development, synaptic organization, neuroimmune tone, and, ultimately, behavioral and cognitive trajectories. A major strength of the current evidence base is that it supports a temporally organized model of transmission. During pregnancy, the placental interface appears to function primarily as a selective metabolic and immunological gatekeeper rather than a route for routine transfer of viable bacteria.^14^ Maternal microbiota-derived metabolites, including SCFAs, tryptophan-related metabolites, and bile-acid intermediates, can nevertheless reach the fetus and influence developmental pathways relevant to neurodevelopment.^15^^,^^92^^,^^129^^,^^131^ At birth, vaginal delivery facilitates transfer of maternal vaginal and fecal taxa, especially taxa such as Bacteroides, Bifidobacterium, and Lactobacillus, that support early immune education and microbiome maturation.^106^^,^^144^ During lactation, the milk microbiome and milk bioactives further shape infant gut colonization and may influence neurodevelopment through microbial, immunological, and metabolic signaling.^91^^,^^151^ Early tactile and environmental exposures, including skin-to-skin contact, add an additional postnatal layer of microbial and neurobehavioral regulation.^152^^,^^157^

At the mechanistic level, the literature increasingly suggests that the developmental relevance of the microbiome lies less in taxonomic presence alone than in microbial function. Recurrently implicated pathways include SCFA production, bile-acid transformation, tryptophan metabolism, inflammatory signaling, and modulation of epithelial and BBB integrity.^92^^,^^108^ This is important because the same microbial species may exert beneficial or detrimental effects depending on strain identity, ecological context, host metabolic state, and timing of exposure. Accordingly, the relationship between parental microbiomes and offspring neurodevelopment should not be interpreted through overly simplified dichotomies of “beneficial” versus “harmful” taxa. Instead, the evidence favors a context-dependent developmental ecology in which maternal eubiosis tends to support immune and neurodevelopmental homeostasis, whereas maternal dysbiosis associated with obesity, metabolic dysfunction, stress, poor diet, or antibiotic exposure may shift metabolite and immune profiles toward inflammatory developmental risk.^43^^,^^78^^,^^89^^,^^201^ The paternal microbiome adds a complementary, though still less well-defined, dimension to this model. Emerging evidence suggests that paternal gut and seminal microbial states may influence offspring development indirectly through sperm-associated epigenetic remodeling, small non-coding RNA cargo, and interactions with the maternal reproductive environment around conception.^12^^,^^13^^,^^188^ Although direct human evidence remains limited, this literature broadens the developmental framework from a maternal-only model to a dual-parent model of microbiome programming. Taken together, the available data support the view that parental microbiomes do not act as isolated compartments, but as coordinated biological systems whose signals intersect during sensitive windows of embryonic, fetal, and early postnatal development.

### 8.2. Translational and clinical implications

The translational relevance of this field lies in the fact that several microbiome-related influences on offspring development are potentially modifiable. Maternal diet, metabolic health, psychological stress, antibiotic exposure, mode of delivery, and breastfeeding practices all affect microbial transfer or microbial signaling during critical windows of neurodevelopment.^82^^,^^89^^,^^91^^,^^169^^,^^175^ This creates an opportunity to move from descriptive microbiome science toward developmental prevention strategies. However, the translational message must remain proportionate to the evidence. At present, the field supports cautious microbiome-informed optimization of perinatal health, but not deterministic prediction or routine clinical manipulation of the microbiome for neurodevelopmental benefit. Among candidate interventions, dietary modulation appears particularly promising because it acts upstream of microbial composition and function. Fiber-rich and polyphenol-rich dietary patterns consistently favor SCFA-producing and anti-inflammatory microbial profiles in both clinical and preclinical settings, whereas high-fat diets are more often associated with dysbiosis, endotoxemia, and altered offspring developmental outcomes.^169^^,^^179^^,^^184^ Probiotics also show potential, especially in relation to maternal metabolic health, infant gut colonization, and milk microbial composition, but their effects are strain-specific and not yet sufficiently standardized for broad neurodevelopmental recommendations.^109^^,^^165^^,^^202^ Similarly, growing evidence around cesarean-associated dysbiosis and microbial restoration strategies is encouraging, but current neurodevelopmental outcome data remain preliminary and do not justify oversimplified clinical claims.^140^

A second translational implication is conceptual rather than interventional: future maternal–fetal and early-life risk assessment should increasingly consider the microbiome as part of a broader developmental systems framework. Microbial signatures alone are unlikely to serve as sufficiently robust biomarkers, but integrated models combining microbial, metabolomic, inflammatory, and clinical variables may eventually improve stratification of pregnancies or infants at elevated neurodevelopmental risk. Importantly, this approach will require much stronger validation across populations and careful attention to confounding by socioeconomic, dietary, obstetric, and environmental variables. A third implication is that translational messaging must distinguish clearly between findings from human cohorts and mechanistic evidence from animal models. Animal studies have been invaluable for establishing causal links between microbial perturbation and offspring brain outcomes, but many pathways remain incompletely demonstrated in humans. Accordingly, preventive strategies should be framed around improving overall perinatal health and microbial resilience rather than promising direct prevention of specific neurodevelopmental disorders. This distinction is essential for both scientific rigor and ethical communication.

### 8.3. Key knowledge gaps and future directions

Despite rapid progress, several major gaps continue to limit interpretation and clinical translation. First, causality remains a central challenge. Many associations between parental microbiome features and offspring neurodevelopment are compelling, but they remain vulnerable to confounding and reverse interpretation. This is especially relevant for concepts such as “dysbiosis,” which are often used descriptively without sufficient mechanistic precision. Future work should more clearly distinguish correlation, mediation, and causation, and should avoid implying that microbiome alterations are necessarily primary drivers rather than markers or amplifiers of broader maternal or paternal physiological states. Second, the field still relies heavily on animal models for mechanistic depth. These models are indispensable, but human and animal evidence are not interchangeable. Human studies often provide ecological validity but limited causal resolution, whereas animal studies provide experimental control but may not fully recapitulate human developmental timing, microbial ecology, or social context. More explicit separation of human-derived and animal-derived conclusions throughout the literature will improve interpretability and prevent overstatement. Third, taxonomic description still exceeds functional understanding. Repeated identification of genera associated with favorable or adverse outcomes is useful, but insufficient. Strain-level resolution, metabolomics, and functional validation are now necessary to determine which microbial products actually mediate effects on the placenta, immune system, gut barrier, BBB, and developing brain. This is particularly important for taxa with context-dependent behavior and for niches such as human milk, where microbial identity alone may not capture developmental relevance. Fourth, paternal microbiome research remains at an early stage. Although the concept of paternal microbial programming is increasingly plausible, evidence is still sparse regarding seminal microbiome composition, its determinants, its interaction with the maternal reproductive tract, and its specific neurodevelopmental consequences in offspring. This area requires careful expansion, particularly in human studies, if the dual-parent framework is to move beyond conceptual novelty into a robust evidence base. Fifth, the field needs better integration across developmental windows and microbial niches. Most studies focus on a single niche, a single time point, or a single exposure. Yet the strongest emerging model is one of sequential and interacting influences across pregnancy, birth, lactation, and infancy. Future studies should therefore prioritize longitudinal mother–infant cohort designs, integration of microbial, metabolomic, and neurodevelopmental data, clear distinction between human and animal-derived evidence, and functional validation of microbiome-derived metabolites. Such designs will be crucial for identifying when parental microbial influences are most developmentally consequential and which interventions are most likely to be effective.

In conclusion, the evidence reviewed here supports a revised view of early-life neurodevelopment in which parental microbiomes contribute to developmental programming through coordinated metabolic, immunological, microbial, and epigenetic pathways. Maternal multi-niche microbial ecosystems remain the principal drivers of early-life exposure, while paternal microbiome-associated effects are emerging as important complementary influences. Across these parental contributions, the microbiome–gut–brain axis provides a biologically plausible framework linking microbial ecology to neurodevelopmental trajectories. At the same time, the field remains in a transitional phase, moving from association-rich description toward mechanism-based and clinically relevant understanding. The most important next step is not simply to catalog more taxa, but to define when, how, and under which conditions parental microbial signals shape the developing brain. Achieving that goal will be essential for transforming microbiome research into credible strategies for early-life neurodevelopmental risk reduction.

## Data Availability

Not applicable.

## Abbreviations

BBB: blood–brain barrier
BMI: body mass index
CPS: capsular polysaccharides
DNA: deoxyribonucleic acid
HMO: human milk oligosaccharides
HPA: hypothalamic–pituitary–adrenal
IDO: indoleamine-2,3-dioxygenase
IL: interleukin
ILA: indole-3-lactic acid
KC: kangaroo care
LPS: lipopolysaccharide
MGBA: microbiota–gut–brain axis
RNA: ribonucleic acid
SCFA(s): short-chain fatty acid(s)
TMAO: trimethylamine-N-oxide
